# Cardiovascular rhythmicity in overweight and obese children

**DOI:** 10.1590/2175-8239-JBN-2022-0138en

**Published:** 2023-07-31

**Authors:** Catarina Pinto-Silva, Ana Correia-Costa, Cláudia Moura, Cláudia Mota, António Guerra, José Carlos Areias, Franz Schaefer, Alberto Caldas Afonso, Elke Wühl, Ana Azevedo, Liane Correia-Costa

**Affiliations:** 1 Centro Hospitalar Universitário de Coimbra, Divisão de Pediatria, Coimbra, Portugal.; 2 Universidade do Porto, Faculdade de Medicina da Universidade do Porto, Divisão de Pediatria, Porto, Portugal.; 3 Centro Hospitalar e Universitário de São João, Divisão de Cardiologia Pediátrica, Porto, Portugal.; 4 Centro Hospitalar Universitário de São João, Serviço de Pediatria, Unidade de Nutrição Pediátrica, Porto, Portugal.; 5 Universidade de Heidelberg, Centro de Pediatria e Medicina do Adolescente, Divisão de Nefrologia Pediátrica, Heidelberg, Alemanha.; 6 Centro Hospitalar Universitário de Santo António, Centro Materno-Infantil do Norte, Unidade de Nefrologia Pediátrica, Porto, Portugal.; 7 Universidade do Porto, Instituto de Ciências Biomédicas Abel Salazar, Porto, Portugal.; 8 Universidade do Porto, Instituto de Saúde Pública, Unidade de Investigação em Epidemiologia, Porto, Portugal.; 9 Universidade do Porto, Faculdade de Medicina, Saúde Pública e Ciências Forenses, Departamento de Educação Médica, Porto, Portugal.

**Keywords:** Pediatric Obesity, Circadian Rhythm, Ultradian Rhythm, Obesidade Infantil, Ritmo Circadiano, Ritmo Ultradiano

## Abstract

**Introduction:**

Obesity is thought to play a role in the disruption of cardiac rhythmicity in obese children, but this is mostly an unexplored field of investigation. We aimed to evaluate the impact of overweight and obesity on circadian and ultradian cardiovascular rhythmicity of prepubertal children, in comparison with normal weight counterparts.

**Methods:**

We performed a cross sectional study of 316 children, followed in the birth cohort Generation XXI (Portugal). Anthropometrics and 24-hour ambulatory blood pressure were measured and profiles were examined with Fourier analysis for circadian and ultradian blood pressure (BP) and heart rate (HR) rhythms.

**Results:**

Overweight/obese children presented more frequently a non-dipping BP pattern than normal weight counterparts (31.5% vs. 21.6%, p = 0.047). The prevalence of 24-hour mean arterial pressure (MAP) and 8-hour HR rhythmicity was significantly lower in obese children (79.3% vs. 88.0%, p = 0.038 and 33.3% vs. 45.2%, p = 0.031, respectively). The prevalence of the remaining MAP and HR rhythmicity was similar in both groups. No differences were found in the median values of amplitudes and acrophases of MAP and HR rhythms.

**Discussion:**

The alterations found in rhythmicity suggest that circadian and ultradian rhythmicity analysis might be sensitive in detecting early cardiovascular dysregulations, but future studies are needed to reinforce our findings and to better understand their long-term implications.

## Resumo

**Introdução:**

Acredita-se que a obesidade desempenhe um papel na desregulação da ritmicidade cardíaca em crianças obesas, mas esse é um campo de investigação ainda pouco explorado. O objetivo deste trabalho foi avaliar o impacto do sobrepeso e da obesidade na ritmicidade cardiovascular circadiana e ultradiana de crianças pré-púberes, em comparação com crianças com peso normal.

**Métodos:**

Realizamos um estudo transversal com 316 crianças, acompanhadas na coorte de nascimentos Geração XXI (Portugal). Foram medidos dados antropométricos e a pressão arterial ambulatorial de 24 horas, e os perfis foram examinados com uma análise de Fourier para ritmos circadianos e ultradianos de pressão arterial (PA) e frequência cardíaca (FC).

**Resultados:**

Crianças com sobrepeso/obesidade apresentaram mais frequentemente um padrão de PA não-dipper em comparação com crianças com peso normal (31,5% *vs*. 21,6%; p = 0,047). A prevalência da pressão arterial média (PAM) de 24 horas e da ritmicidade da FC de 8 horas foi significativamente menor em crianças obesas (79,3% *vs*. 88,0%; p = 0,038 e 33,3% vs. 45,2%; p = 0,031, respectivamente). A prevalência das restantes ritmicidades da PAM e da FC foi semelhante em ambos os grupos. Não foram encontradas diferenças nos valores medianos das amplitudes e acrofases dos ritmos de PAM e FC. Discussão: As alterações encontradas na ritmicidade sugerem que a análise da ritmicidade circadiana e ultradiana pode ser sensível na detecção de desregulações cardiovasculares precoces, mas são necessários novos estudos para reforçar nossos achados e entender melhor suas implicações a longo prazo.

## INTRODUCTION

The prevalence of childhood overweight and obesity has risen substantially in less than one generation worldwide^1^, increasing from 0.7 to 5.6% in girls and from 0.9 to 7.8% in boys aged 5–19 from 1975 to 2016^2^. The excess weight is linked to several deleterious consequences, not only affecting childhood health status and wellbeing^3^, but also increasing the risk of cardiovascular disease in adulthood^4,5^.

In children, there is evidence that obesity is one of the most important modifiable risk factors for hypertension. 24-hour ambulatory blood pressure monitoring (ABPM) has been shown to be more accurately related with target-organ damage and a better predictor of cardiovascular risk than office blood pressure (BP) measurements^6,7^. 

Nonetheless, besides being considered the gold standard for BP evaluation and diagnosis of hypertension in the pediatric population^8–10^, the information content of ABPM is usually not fully explored^8^. Numerous mathematical approaches have been applied to study the data generated by ABPM, such as chronobiological cosinor analysis^11^. The Fourier analysis can be used to describe complex, asymmetrical, and multiphasic BP profiles by simultaneously applying several cosine functions^12^, combining several rhythms, and allowing a more detailed and flexible description of BP and heart rate (HR) over the 24-hour period^11^.

Ultradian rhythmicity, i.e., significant variations in cardiovascular rhythm in periods shorter than 24 hours, was described in adults^13^ and more recently also in healthy children, in whom 6-, 8-, and 12-hour cardiovascular rhythms can be identified^11^. The biological mechanism underlying BP rhythmicity is still largely elusive, but in some disease conditions there seems to exist a rhythmicity disruption^14–17^.

In children with chronic kidney disease, circadian and ultradian cardiovascular rhythmicity were found to be blunted and quantitatively associated with kidney function and proteinuria^14^. Blunted ultradian BP rhythm was also found in children with ambulatory hypertension and white coat hypertension^16^ and in prepubertal children born small-for-gestational age, independent of the presence of hypertension^15^. 

In the pediatric setting, only a few studies evaluated the impact of obesity on cardiovascular ultradian rhythmicity with contradictory findings^16,17^. We hypothesize that circadian and ultradian rhythmicity may be sensitive indicators of an underling cardiovascular dysregulation, already present in overweight young children. Thus, in the present study we aimed to evaluate the impact of overweight and obesity on the circadian and ultradian cardiovascular rhythmicity of prepubertal children, in comparison with their normal weight peers. 

## METHODS

**Figure 1.**
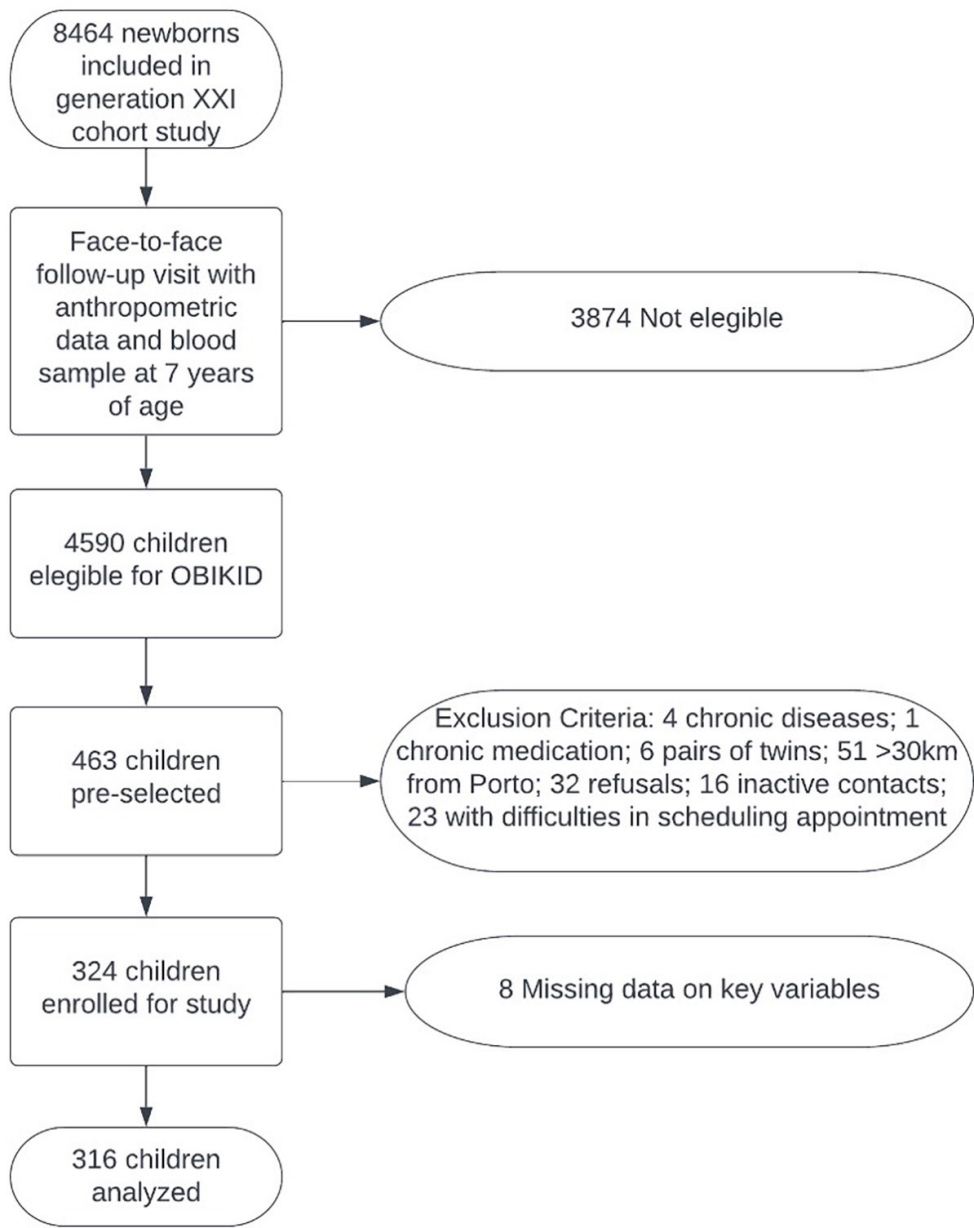
Flowchart depicting the methodology used for participant selection.

We analyzed a group of children aged 8 and 9 years that have been followed since birth in a previously established cohort study (Generation XXI, Porto-Portugal)^18^. Selection of participants from that cohort is depicted in Figure 1. A total of 316 children (166 with normal weight, 150 obese or overweight) were finally included, which provides a statistical power above 93% to detect a difference in the prevalence of 24-h MAP rhythm of at least 15% between non-overweight and overweight/obese groups^17^.

The study visits took place at the Department of Clinical Epidemiology, Predictive Medicine and Public Health, Faculty of Medicine of University of Porto. Anthropometric and general physical examination were performed according to standard procedures as previously reported^19^. Body mass index (BMI) and BMI-for-age values were used to categorize children into the following groups: non-overweight (BMI-SDS (standard deviation score) ≤ +1) and overweight/obesity (BMI-SDS > +1, including children with overweight (BMI-SDS > +1 and obesity (BMI-SDS > +2), according to the World Health Organization reference data^20^.

ABPM was performed in all children with a portable non-invasive oscillometric BP recorder (Spacelabs Healthcare^®^, model 90207, Snoqualmie, Washington, USA) in the non-dominant arm and with a cuff size appropriate to the child’s arm circumference. BP measurements were automatically evaluated at 20-minute intervals during the day and at 30-minute intervals at night. The nighttime period was defined from 00:00 PM to 06:00 AM and daytime from 08:00 AM to 08:00 PM. For quality assurance, a minimum monitoring of 24 hours with gaps of less than 2 hours was required. The readings were used to calculate 24-h, daytime, and nighttime mean arterial pressure (MAP), systolic (SBP) and diastolic BP (DBP) using the SpaceLabs^®^ software. Using the least mean square method, the standard deviation scores for BP values were calculated and hypertension was defined using published reference values of the German Working Group on Pediatric Hypertension derived from healthy mid-European children and adolescents (ABPM reference population)^21^. Absence of dipping was considered when MAP dropped during nighttime less than 10% of the corresponding daytime MAP. 

ABPM profiles were examined by Fourier analysis for the prevalence of circadian (24 h) and ultradian (12, 8, and 6 h) BP and heart rate (HR) rhythms. For each rhythm identified with a p < 0.05 by least-square analysis, a MESOR (median value between the lowest and highest value of the fitted curve), an amplitude (half the distance between the maximum and minimum values of the cosine curve, in mmHg), and an acrophase (time of the maximum, in hours after midnight) were calculated. Fourier analysis was performed by SAS 9.3 Software package (SAS Institute Inc., Cary, NC, USA). The prevalence of circadian and the defined ultradian rhythmicity were compared to the published prevalence data of the ABPM reference population^21^.

The ObiKid study was approved by the Ethics Committee of Centro Hospitalar Universitário de São João, Porto – Portugal and Faculty of Medicine of the University of Porto and by the Portuguese Data Protection Authority. It complies with the Helsinki Declaration and the current national legislation. Written informed consent from parents (or their legal substitute) and verbal assent from children was obtained regarding the collection of information and biological samples.

Statistical analysis was performed using IBM SPSS Statistics, Version 24.0 (Armonk, NY). Data are presented as mean and standard deviation (SD) or, if skewed, as median with percentiles (percentile 25 (P25)-percentile 75 (P75)). Differences between groups were evaluated using Chi-square test for dichotomous variables and Mann-Whitney test for continuous variables. Logistic regression models were fitted to estimate the influence of 24-h MAP levels in the prevalence of different rhythmicity. Significance was determined at p < 0.05.

## RESULTS

A total of 316 children (52.8% male) with a mean (SD) age of 8.8 (0.2) years were included in the present analysis. Demographic and anthropometric characteristics and 24-h ABPM values are shown in Table 1 by classes of BMI. The median (P25-P75) ABPM values, 24-h, daytime and nighttime MAP, and SBP SDS were significantly higher in the overweight/obese children. No differences were found between the groups regarding the absolute values (in mmHg) of 24-h, daytime, or nighttime MAP, SBP, or DBP. Among overweight/obese children, hypertension was diagnosed in a higher percentage, but this difference was not statistically significant.

**Table 1.** Anthropometric and clinical characteristics of non-overweight (n = 166) and overweight/obese (n = 150) participants

	Non-overweight^1^	Overweight/obese^1^	*p*
	n = 166	n = 150	
**Demography and anthropometry**			
Age (months)	105 (102–108)	106 (103–108)	0.275
Male sex	84 (50.6%)	83 (55.3%)	0.400
Weight (kg)	27.7 (25.2–29.9)	37.2 (34.2–42.6)	0.000
Height (cm)	131.2 (128 –134.8)	135.4 (131.3–139.3)	0.000
BMI z-score	0.1 (–0.4–0.6)	1.9 (1.5–2.5)	0.000
**24-h ambulatory blood pressure**			
24-h MAP (mmHg)	81.7 (78.9–84.8)	81.4 (79.0–84.5)	0.367
SDS	0.3 (–0.2–0.9)^*^	0.5 (0.0–1.1)^*^	0.035
Daytime MAP (mmHg)	85.2 (81.9–89.3)	84.8 (81.7–88.1)	0.420
SDS	–0.0 (–0.5–0.6)	0.2 (–0.4–0.7)^*^	0.035
Nighttime MAP (mmHg)	73.5 (70.0–77.6)	73.4 (70.1–76.0)	0.538
SDS	0.6 (0.0–1.0)^*^	0.7 (0.2 –1.2)^*^	0.032
24-h SBP (mmHg)	112.3 (108.1–117.0)	112.7 (107.9–116.7)	0.831
SDS	0.5 (–0.1–1.2)^*^	0.8 (0.3 –1.4)^*^	0.001
Daytime SBP (mmHg)	116.3 (111.9–121.5)	116.2 (111. –121.5)	0.842
SDS	0.4 (–0.2–1.0)^*^	0.7 (–0.0–1.3)^*^	0.008
Nighttime SBP (mmHg)	103.5 (98.4–109.0)	104.3 (98.5–107.7)	0.861
SDS	0.7 (–0.1–1.2)^*^	0.9 (0.4–1.4)^*^	0.000
24-h DBP (mmHg)	67.1 (64.1–70.5)	66.0 (63.2–69.0)	0.034
SDS	0.0 (–0.5–0.7)	0.1 (–0.5–0.8)^*^	0.468
Daytime DBP (mmHg)	71.2 (67.5–74.6)	70.0 (66.6–73.6)	0.107
SDS	–0.3 (–0.8–0.3)^*^	–0.3 (–0.9–0.3)^*^	0.932
Nighttime DBP (mmHg)	57.5 (55.0–61.7)	57.2 (52.7–60.0)	0.072
SDS	0.3 (–0.4–0.7)^*^	0.3 (–0.3–0.9)^*^	0.268
24-h HR (bpm)	82.1 (77.8–86.2)	81.3 (76.9–86.2)	0.356
Daytime HR (bpm)	86,4 (81.1–92.2)	85,5 (80.3 –91.1)	0.366
Nighttime HR (bpm)	72.0 (67.0–77.1)	71.7 (66.3–77.1)	0.949
Absence of dipping^2^	35 (21.6%)	47 (31.5%)	0.047
Hypertension^3^	12 (7.2%)	18 (12.0%)	0.149

Data are reported as median (P25–P75) or n (%). BMI: body mass index; MAP: mean arterial pressure; SDS: standard deviation score; SBP: systolic blood pressure; DBP: diastolic blood pressure; HR: heart rate.

^*^Standard deviation score (SDS) significantly different from ABPM reference population values.

^1^The BMI classes are according to the WHO classification for BMI z-score values [38].

^2^Absence of dipping was defined as a fall in MAP during nighttime of <10% of the corresponding daytime BP.

^3^Hypertension was defined using published reference values of the German Working Group on Pediatric Hypertension [21].

Compared to the ABPM reference population^21^, 24-h, daytime, and nighttime SBP SDS, and 24-h and daytime MAP SDS values were significantly higher in the overweight/obese as well as in the non-obese study cohort (except for 24-h DBP SDS and daytime MAP SDS in non-obese that did not differ), while DBP SDS values were lower (daytime) or did not differ (24-h and nighttime). The largest divergences between the study cohorts and the reference population were found for nighttime BP. The higher nocturnal blood pressure levels might also explain the overall higher prevalence of nocturnal non-dippers in the study cohorts.

The prevalence of MAP and HR rhythmicity in non-overweight and in overweight/obese children is presented in Table 2. The prevalence of 24-h MAP and 8-h HR rhythmicity was significantly lower in obese children (79.3% vs. 88.0%, p = 0.038 and 33.3% vs. 45.2%, p = 0.031, respectively), while the prevalence of the remaining MAP and HR rhythmicity was similar in both groups. All ultradian rhythmicity were significantly more prevalent in the study cohorts compared to the reference group. In logistic regression models, the presence of each BP rhythmicity was found to be independent of 24-h MAP values.

**Table 2.** Prevalence of mean arterial pressure and heart rate rhythmicity in non-overweight and overweight/obese participants

	Non-overweight^1^	Overweight/obese^1^	*Healthy Controls* ^2^	*p*
**Prevalence of MAP rhythms**				
24-h rhythmicity	146 (88.0%)	119 (79.3%)	90%	0.038
12-h rhythmicity	91 (54.8%)	85 (56.7%)	28%	0.741
8-h rhythmicity	91 (54.8%)	68 (45.3%)	34%	0.092
6-h rhythmicity	65 (39.4%)	57 (38.0%)	18%	0.833
**Prevalence of HR rhythms**				
24-h rhythmicity	139 (83.7%)	124 (82.7%)	96%	0.800
12-h rhythmicity	88 (53.0%)	80 (53.3%)	36%	0.924
8-h rhythmicity	75 (45.2%)	50 (33.3%)	30%	0.031
6-h rhythmicity	64 (38.6%)	54 (36.0%)	17%	0.639

Data are reported as percentage.

MAP: mean arterial pressure; HR: heart rate.

^1^BMI classes are according to the WHO classification for BMI z-score values [38].

^2^Data derived from healthy reference cohort [21].

**Figure 2.**
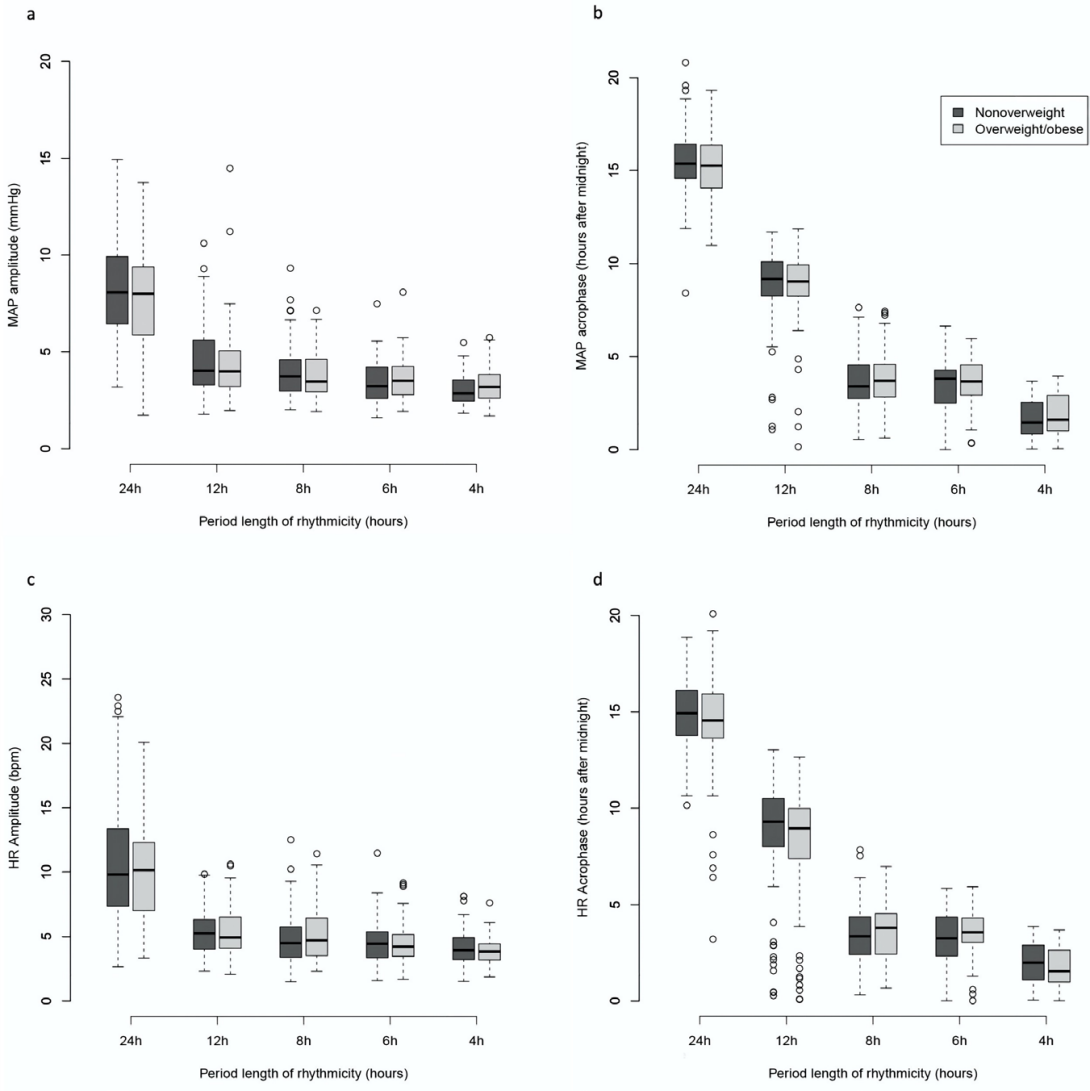
Distribution of amplitudes and acrophases of mean arterial pressure (MAP) and heart rate (HR) by classes of body mass index (BMI: non-overweight and overweight/obese). MAP amplitudes of circadian and ultradian rhythms (panel a.); MAP acrophases of circadian and ultradian rhythms (panel b.); HR amplitudes of circadian and ultradian rhythms (panel c.); HR acrophases of circadian and ultradian rhythms (panel d.). The amplitude and acrophase data are expressed as medians and 25 and 75 percentiles. The BMI classes are according to the WHO classification for BMI z-score values [38]. bpm: beats per minute.

No differences were found in median values of 24-h, 12-h, 8-h, and 6-h amplitudes and acrophases of MAP and HR rhythmicity between the non-overweight and overweight/obese groups (Table 3 and Figure 2). Comparing the study cohorts to the ABPM reference population, MAP amplitudes were flattened and acrophases delayed (Table 3). On average, MAP amplitudes were 1.7 to 2.4 mmHg flatter and acrophases were delayed by 1.2 to 1.8 hours for the different ultradian rhythmicity. Similar effects were seen for HR amplitudes and acrophases. 

**Table 3.** Median amplitudes and acrophases of mean arterial pressure and heart rate in non-overweight and overweight/obese participants

	Non-overweight^1^	Overweight/obese^1^	*Healthy Controls* ^2^	*p*
**MAP**				
24-h amplitude	8.1 (6.4–9.9)	8.0 (5.7–9.4)	10.1 (8.1–12.4)	0.501
24-h acrophase	15.4 (14.6–16.4)	15.3 (14.0–16.4)	13.9 (13.1–15.0)	0.153
12-h amplitude	4.0 (3.3–5.6)	4.0 (3.2–5.1)	5.9 (4.8–7.2)	0.571
12-h acrophase	9.2 (8.3–10.1)	9.0 (8.2–10.0)	7.8 (6.4–8.7)	0.789
8-h amplitude	3.7 (3.0–4.6)	3.5 (2.9–4.6)	6.1 (5.1–7.5)	0.377
8-h acrophase	3.4 (2.7–4.6)	3.7 (2.8–4.6)	2.1 (1.3–3.1)	0.438
6-h amplitude	3.2 (2.6–4.2)	3.5 (2.8–4.3)	5.2 (4.4–6.6)	0.379
6-h acrophase	3.8 (2.4–4.3)	3.7 (2.9–4.6)	2.0 (1.5–3.0)	0.530
**HR**				
24-h amplitude	9.8 (7.3–13.4)	10.1 (7.0–12.3)	15.4 (12.3–19.1)	0.346
24-h acrophase	14.9 (13.8–15.9)	14.5 (13.6–15.9)	13.8 (12.9–14.7)	0.188
12-h amplitude	5.2 (4.0–6.3)	4.9 (4.0–6.5)	7.7 (6.0–9.9)	0.922
12-h acrophase	9.2 (8.3– 0.1)	9.0 (8.2–10.0)	8.4 (7.4–9.4)	0.789
8-h amplitude	3.7 (3.0–4.6)	3.5 (2.9–4.6)	7.7 (5.9–9.7)	0.377
8-h acrophase	3.4 (2.7–4.6)	3.7 (2.8–4.6)	1.8 (1.0–3.8)	0.438
6-h amplitude	3.2 (2.6–4.2)	3.5 (2.8–4.3)	6.4 (5.4–8.1)	0.379
6-h acrophase	3.8 (2.4–4.3)	3.7 (2.9–4.6)	2.0 (1.5–2.8)	0.530

Data are reported as median (P25 - P75).

MAP: mean arterial pressure; HR: heart rate.

^1^The BMI classes are according to the WHO classification for BMI z-score values [38].

^2^Data derived from healthy reference cohort [21].

## DISCUSSION

In the present study, we found that overweight/obese children presented a non-dipping BP pattern and lower prevalence of 24-h MAP and 8-h HR rhythmicity more frequently than non-overweight children. These findings might represent non-negligible differences in circadian and ultradian rhythmicity associated with obesity status. The prevalence of the remaining rhythms studied was similar between groups, and we could not find statistically significant differences regarding the amplitudes and acrophases of rhythms between non-overweight and overweight/obese children. Interestingly, both groups differed from the reference population, which consisted of healthy mid-European children, with respect to prevalence of ultradian BP rhythmicity and MAP and HR amplitudes and acrophases analyzed. 

Altered circadian variability in obese children has been described in previous studies^9,22,23,24^ and, mainly in adult studies, it has been linked to various adverse outcomes, such as increased risk of cardiovascular events^25,26^. In line with previous studies, in our study, we observed a higher frequency of non-dipping pattern in the overweight/obese children. Nonetheless, we aimed to explore in detail not only circadian variability but also ultradian rhythmicity derived from ABPM studies, as this is still an unexplored field, especially when considering children with specific pathologies or comorbidities.

Hadtstein et al. reported the existence of 6-, 8-, 12-h, and circadian cardiovascular rhythmicity in the majority of healthy children but the meaning and the causes of this rhythmicity have not yet been explored in many studies^11^, and conflicting data has been reported in the literature. In our study, we found a lower prevalence of circadian rhythmicity among overweight/obese children and an increase in ultradian rhythmicity in both study groups. Before our study, Saner et al. were the first to explore BP rhythmicity changes in association with obesity in children and their findings are consistent with ours, describing a lower prevalence of 24-h and 6-h MAP rhythmicity among obese children^17^. Other studies have previously explored BP rhythmicity in other specific settings; Wuhl et al. found that in pre-pubescent children with chronic kidney disease, circadian HR and BP rhythmicity was less prevalent than in healthy controls, while the prevalence of ultradian 12-h MAP and HR rhythmicity was markedly higher in these children. In this research, a trend was found towards a higher prevalence of 8-hour and 6-hour MAP and HR rhythmicity in the chronic kidney disease prepubescent and pubescent children compared with control subjects, but this was significant only for 8-hour MAP rhythmicity in the group of pubescent children^14^. Another group reported higher prevalence of 12-h rhythmicity in children with white coat hypertension and ambulatory hypertension but failed to identify any strong and independent effect of obesity on BP rhythmicity^16^. 

An altered BP rhythmicity has usually been interpreted as an early sign of cardiovascular health impairment in specific groups of patients. We found a higher frequency of some ultradian rhythms in overweight and obese children but the prevalence of rhythmicity was found to be independent of BP levels, as previously stated by other authors in the same setting^17^. In chronic renal disease patients, higher ultradian rhythmicity was interpreted as a possible consequence of higher blood pressure^14^. However, in this group of patients, no consistent relationship between ultradian BP amplitudes and MAP levels was found, indicating that ultradian amplitude changes may be more directly related to kidney disease, rather than from hypertension.

Considering the results of previous studies, we would expect some blunting of BP and HR amplitudes and a delay in acrophases in overweight and obese children. Indeed, compared to the ABPM reference population, overweight children had flattened MAP and HR amplitudes and acrophases. However, we could not find any statistically significant differences between the overweight/obese and the non-overweight groups regarding these values. This might indicate that there may be other risk factors for early cardiovascular alterations besides overweight and hypertension in the non-obese Portuguese control group.

Saner et al. reported that obese subjects showed lower amplitudes of 24-hour HR and higher 24-hour MAP and HR acrophases^17^. In that study, obese children were slightly older than in our study, with a median age of 11.6 years in the obese group. Previous studies have reported that some changes in cardiovascular rhythmicity are expected to occur around puberty^11^, which might explain in part why we did not find more pronounced differences in our study, which only included prepubertal children aged 8 and 9 years. Moreover, we included not only obese, but also overweight children, while Saner et. al only included obese children, which might have increased the chance of significant differences. Nonetheless, in our study, we found no differences when comparing obese and overweight children or obese and non-overweight children. 

While circadian rhythmicity appears to be generated in the hypothalamus, ultradian rhythmicity seems to rely more on the sympathetic drive^14,16,17^. While hypertension is considered a multifactorial and polygenic trait^27^, there is evidence that the sympathetic nervous system is overactivated^28^ in obesity and this is recognized as a major actors in the development and maintenance of hypertension in the setting of obesity. Adiposity is known to stimulate the activation of SNS, particularly through visceral adipose tissue^29^. Considering this, disruptions in ultradian rhythmicity might be used as non-invasive markers of early subclinical changes in cardiac function in obese children. Future studies need to confirm that these changes may lead to an increased cardiovascular risk and mortality later in life, as has already been hypothesized^30,31^.

The main limitation of our study is related to the cross-sectional design. Clearly, a long-term follow-up of these children would be of utmost importance to insure a better comprehension of the direction and prognostic value of the variables studied. A major strength of our work is that the study of the ultradian cardiovascular rhythmicity in association with obesity is an innovative area of research. Analyzing a large sample of prepubertal children homogenous in terms of age allowed us to study cardiovascular rhythmicity in young individuals avoiding the issue of secondary pathology related to target organ damage and comorbidities often encountered in ageing populations.

In conclusion, our study covers a largely unexplored area. While our findings suggest that circadian and ultradian rhythmicity might be sensitive indicators of early cardiovascular dysregulations, prospective observation is required to confirm the long-term implications of these early alterations for target organs and morbidities. Finally, we consider that the study of circadian and ultradian cardiovascular rhythmicity is valuable and that future studies might help to establish this detailed ABPM analysis in the assessment of early cardiovascular morbidity.
